# Light Emitted Diode on Detecting Thin-Film Transistor through Line-Scan Photosensor

**DOI:** 10.3390/mi12040434

**Published:** 2021-04-14

**Authors:** Fu-Ming Tzu, Jung-Shun Chen, Shih-Hsien Hsu

**Affiliations:** 1 Department of Marine Engineering, National Kaohsiung University of Science and Technology, Kaohsiung 80543, Taiwan; 2 Department of Industrial Technology Education, National Kaohsiung Normal University, Kaohsiung 80201, Taiwan; jschen@nknu.edu.tw; 3 Department of Electrical Engineering, Feng Chia University, Taichung 40724, Taiwan; shihhhsu@fcuoa.fcu.edu.tw

**Keywords:** light-emitted diode, thin-film transistor, photosensor, spectrometer, optical inspection

## Abstract

This paper explores the effectiveness of the white, red, green, and blue light emitted diodes (LEDs) light sources to detect the third layer of the electrode pixel and the fourth layer of the via-hole passivation on thin-film transistors. The time-delay-integration charge-coupled device and a reflective spectrometer were implemented in this experiment. The optical conditions are the same, as each light source and the digital image’s binary method also recognize the sharpness and contrast in the task. Consequently, the white and the blue LED light sources can be candidates for the light source for the optical inspection, especially for monochromic blue LED’s outperformance among the light sources. The blue LED demonstrates the high spatial resolution and short wavelength’s greater energy to trigger the photosensor. Additionally, the metal material has shown a tremendous responsibility in the photosensor with 150 Dn/nj/cm^2^ over the sensibility. The mercury ^198^Hg-pencil discharge lamp emits the stable spectral wavelength to significantly calibrate the spectrometer’s measurement.

## 1. Introduction

A light-emitting diodes (LEDs) has become a popular light source over the past decades. Its applications are found across various fields, especially optical inspection. The optical inspection system is similar to a keeper—to detect the defect of a thin film. Once an acquired image is of low quality, it is challenging to deliver the required information. The optical system has three main key components: line-scan charge-coupled device, lenses, and light sources. The three subsystems can be mutually independent, but the most ignorant and influential is the light sources. The appropriate light can reflect the required image characteristics. Presently, the task utilizes the white (rich-blue), red, green, and blue light LEDs to detect the thin-film transistor, whose working voltage is 3 V, and it emits light with an individual brightness. The brightness can be adjusted by voltage (or current) at 250 lumens per watt. Furthermore, the LEDs also have a resistance to shock and vibration and have a long lifespan of 30,000 h. Various materials on LEDs may generate photons with different energies, thereby controlling the wavelength of light emitted in the spectrum of color in that range.

As a display demand moves forward, the thin-film transistor (TFT) becomes a bottleneck in the process and cannot guarantee high quality. In contrast, a TFT uses a semiconductor process, a type of field-effect diode (FET) that switches the electronic flow toward the electrode pixel. However, if the defect adhered to the thin-film transistor would make the electronic signal unstable and have uncertainty in transferring [1,2]. Its production involves depositing various thin films on the substrate in the active layers, including the dielectrics and metal electrodes, which deposit a thin film on the substrate that is fabricated by the backchannel etching process: five-layer processes consist of a gate metal as the first layer, secondly dielectric/channel/n^+^, the third layer is the source/drain metal, the fourth layer is silicon nitride passivation and via-hole, and the final layer is an indium tin oxide (ITO) electrode. The copper metal’s fast electrical conductivity and high transmittance of the thin film can enhance the TFT array’s color rendering.

Most TFTs are made of hydrogenated amorphous silicon (a-Si: H) as the primary material because its energy level is smaller than that of single-crystal silicon. With progress in manufacturing, the TFT resolution tends to be high-definition [3,4] at a comprehensive view illumination coupled to the ultra-high definition and beyond. Thus, the electrode pixel in TFT approaches a smaller and smaller dimension. Recently, low-temperature poly-silicon (LTPS) is one of the most popular technologies in the mainstream display trends of thin-film transistor liquid crystal display (TFT-LCD) manufacturing. The most significant difference from traditional amorphous silicon displays is that LTPS has a faster response speed and higher brightness [5].

In the literature review, Luo et al. (2020) [6], using LTPS, enhanced the optical property’s transmission and chromaticity on the fourth layer of the TFT array. The SiO_x_N_y_ as a passivation layer increases the transmittance. As a result, the task improved 6% in optical transmittance and 18‰ in chromaticity for TFT arrays. Rodriguez-Zamora et al. (2021) [7] utilized the three metals, gold, silver, and copper as the bare nanoparticles used to measure the wavelength spectrum. The emergence of chiroptical activity is found in the wavelength range of 250–400 nm. Ibrahim and Abdughani (2020) [8] estimated the spectral response of the Cr_2_O_3,_ which is an influential band between 310 and 400 nm. It is estimated to reveal a short wavelength spectrum for metal characteristics, including copper, silver, gold, and a combination material chromium oxide (Cr_2_O_3_) thin films. The indication is the most efficient of the chromium emission in the ultraviolet range. Yuan et al. (2018) [9] took a Ce: YAG transparent ceramic excited by a blue-laser diode to address a white light (blue-laser diode mixing) composition of the wavelength using the transmittance light to measure its spectrum phosphor ceramic. The spectrum indicated a wavelength of 565 nm and a width of 200 nm when blue-laser light was converted to yellow light.

The literature reviews made an extraordinary contribution to the optical measurement and wavelength distribution, but the gray distribution was not addressed.

This paper aims to distinguish the LED light source with different wavelengths to detect the TFT electrode pixel engaged reflective spectrometer, engaged with the gray distribution. The image is acquired by multiple line-scan photosensors and utilizes an automatic data acquisition program (ADAP) to approach its gray distribution of the digital image.

## 2. The Principle of Gray Level and Geometrical Optics

The gray level has the most potential as an analyzer of detectability among digital images. Any morphologic defect adhered onto the thin-film transistor using the various gray levels can enhance the characteristics. The gray-level threshold indicated the defect image has nowhere to hide; thus, the so-called binary image is the performance of black and white. However, the color image converts to a grayscale image, and a grayscale value is set for each pixel in the picture, known as the threshold scale. Pixels whose grayscale are more significant than the threshold are converted to white pixels, and pixels whose grayscale are less than the threshold convert to the black pixel. After conversion, a binary image can be obtained. Through the binarization process, some information is hidden in the image or it is challenging to effectively detect and reveal. Once the image is acquired and digitized, the basic unit is a pixel and each pixel has specific coordinates and corresponds to the object. The pixel value is generally called a gray level. The larger the degree level, the higher the brightness. In this task, the gray interval has 256 grays to analyze the image between 0 and 255. Thus, it is an intricate image to simplify, and the reticle cutting image is commonly used to detect a smooth surface. Experimentally, the threshold of *m*, the criterion of binarization as shown in Equation (1), is the image grayscale *m* [10].

(1) $$m=\sum_{i=1}^{n}f\left(x,y\right)$$

The input image is *f*, *n* is the number of all pixels, the pixel’s gray value is *f* (*x*, *y*) with respect to coordinates (*x*,*y*). Once the gray value is lower than *m* of the gray value, the task chooses for it to be 0. On the other hand, once the image’s gray value is higher than the division value *m*, the choice becomes 1. Experimentally, gray level places an appropriate threshold from 0 to 255, 50 to 150, 60 to 140, 70 to 130, 80 to 120, and 90 to 110, respectively. Whereas there are six images compared in their acquired quality, the gray level task will be discussed later.

Moreover, an optical resolution of the acquired image depends on the working distance (*WD*). Either a more satisfactory or a rougher image resolution based on geometrical optics, thereby, more refined and higher resolution and vice versa. The *WD* indicated the distance between the objective and focused surface-based thin-lens maker’s formula. A coarse optical resolution indicates a large magnification field of view (FoV). In geometrical optics, the working distance of the proper optical resolution is shown in Equation (2) [11]:

(2) $$WD=f*\left(\frac{M+1}{M}\right)+f*\left(M+1\right)+\bar{HH^{*}}$$

where *f* is the focal point, *M* is the magnification between the object and subject and $\bar{HH^{*}}$ represents the lens’s thickness. While scanning is performed, a signal capacitor array triggers the photon electronic current on the photoactive region. Each capacitor accumulates electron charges in proportion to the light intensity. Through signal amplification and decoding devices, the quality of the image is dependent on the charge-couple device.

The depth of field (*DoF*) is a critical issue to perform the quality of the scanning image where the distance is between the photosensor and object. In contrast, the optical parameter is determined by object distance, the focal length, the aperture value of the lens, and the *DoF*. The *DoF* is the relatively short-range before and after the camera focus. As a result, the *DoF* image is clear; however, the image before or after the *DoF* range is blurry. The *DoF* of the formula expresses by Equation (3) [12] is shown below:

(3) $$DoF=\frac{sf^{2}}{f^{2}-Ac(s-f)}-\frac{sf^{2}}{f^{2}+Ac(s-f)}$$

where *A* is aperture number, *c* is the circle of confusion, *f* is focal length, *s* expresses the subject distance, and *DoF* is the mean of the depth of field. In this experiment, the acquired images above optical parameters take each image, except for light sources. Moreover, the standard deviation, σ (sigma), illustrated by Equation (4), typically quantifies the accuracy of the measurements, where $\bar{p}$ is the mean value of the dataset $\left\{p_{1},p_{2}\ldots p_{n}\right\}$, and n is the number of samples. On the other hand, the standard deviation, σ (sigma), illustrated by Equation (4), typically quantifies the accuracy of the measurements; where $\bar{p}$ is the mean value of the dataset $\left\{p_{1},p_{2}\ldots p_{n}\right\}$, and n is the number of samples.

(4) $$\sigma =\sqrt{\frac{1}{n-1}\sum_{i=1}^{n}{\left(p_{i}-\bar{p}\right)}^{2}}$$

The lighting tends to affect the acquired image as the various wavelengths project on the TFT experimentally. The light-emitting diode emits the light through the composed materials by trivalent and pentavalent elements. When a voltage is applied to the photodiode at the positive and negative ends at this junction, the current passes, it will combine electrons with holes and emit light based on the different material doping. The wavelength and color are related to semiconductor materials and the elemental impurities deliberately incorporated.

## 3. A Topology of Optical Architecture on Machine Vision

**Figure 1:**
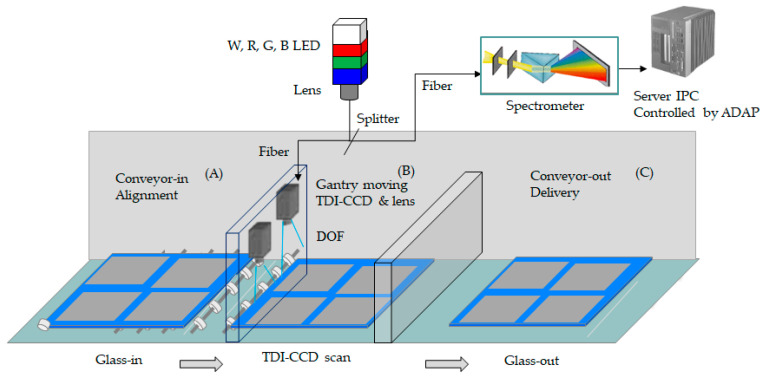
A sequent process of the thin-film transistor (TFT) glass in the architecture of automatic optical inspection. In which (**A**) is the alignment and conveyor-in, (**B**) is the captured image site, and (**C**) is the conveyor-out.

The experiment is carried out at 10 class levels in the clearing room using the glass 6th generation, dimension size 1800 mm × 1500 mm, manufactured by Corning Inc. (Corning, NY, USA). The optical architecture of the automatic optical inspection detected the substrate illustrated in Figure 1. The systemic components included the white, red, green, and blue light-emitting diodes with monochromatic light to TDI-CCD (time delay and integration-charge couple device, manufactured by Teledyne DALSA Inc, Waterloo, ON, Canada) individually. Simultaneously, the automatic data acquisition program (ADAP) controls the optoelectronic motion, mechanism, and gray level analysis. The multiple optical fibers along with the outer metal jacket connect the lighting-focused lens at the coherence light transmitted by Snell’s law [13,14]. The total internal reflection is between the core and cladding due to the high refraction. The optical splitter separates the coherent light to enter the spectrometer and allows for individual visual inspection.

Moreover, the bending angle in the optical fiber might be a concern so that the fiber has less possible motion at the rack. Simultaneously, the server motor drives the gantry to attach the TDI-CCD, automatically acquiring the image. The sequence follows the glass-in with alignment, the light source engaged with TDI-CCD line scan, and the glass-out in the experiment. The scanning time is a crucial matter to determine the mass production throughput. In comparison, the total cycle time takes 160 s for this task.

Figure 1 presents where the glass enters the zone and becomes ready for inspection, the sample needs alignment, and the clamp locks to fix the model in order to accurately coordinate with a pneumatic system. The test-platform design for the pneumatic elasticity prevents the vibration for each foundation of the machine vision. That platform is made of a thickness of 200 mm granite material to have exceptional planarity. The glass delivers to the site for alignment and ready for scanning in Figure 1A. The TFT panel attached is in Figure 1B and automatic optical inspection of TDI-CCD links with a monochromatic LED. Finally, the glass-out is in Figure 1C. The typology consists of the three sections of Figure 1A–C.

On the other hand, the TDI-CCD-engaged coherence-light illuminates the sample. The TDI-CCD is a multiple scanning photosensor used to perform the image quality at a low-light intensity, enhancing the line scan sensitivity. Whereas, there is a new type of CCD with an area array structure and linear array output. The multiple scanning functions of the photosensor outperform an ordinary linear array CCD. The TDI-CCD takes the imaging in low-light environments and has less effect on the scanning speed.

Furthermore, TDI-CCD has the outstanding feature of obtaining high sensitivity without skipping a beat to sacrifice spatial resolution and working speed. The photosensor indicates a wide range of application prospects in the high-speed and low-light fields. This camera uses the model H.S. 12k TDI-CCD experimentally. Specification of Piranha H.S. 12k engaged a fast 90-kHz line rate. The pixel size is at 5.2 μm (H) × 5.2 μm (V). While connected with the magnification lens, the TDI-CCD line scan optical resolution shrinks at 1 μm and even lower 0.5 μm if the magnified lens is installed on the photosensor.

**Figure 2:**
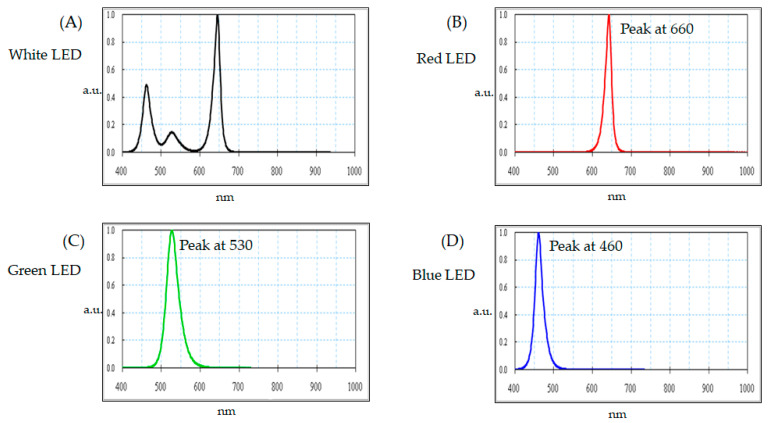
The wavelength distribution at (**A**) white, (**B**) red, (**C**) green, and (**D**) blue light-emitted diodes (LEDs) spectrum, respectively.

The experiment utilizes the white, red, green, and blue LEDs to measure their optical spectrum of the wavelengths in Figure 2A–D, respectively.

The red LED is typically manufactured from aluminum, indium, gallium, and phosphorus, combining all four functional materials—tetra-elements. The central peak of the wavelength of the red LED distributes at 660 ± 2 nm experimentally. The green LED is manufactured using three kinds of functional materials: indium, gallium, and nitride, which are tri-elements, and the central peak is at 530 ± 2 nm. Two or three available materials are made of the blue LED: indium, gallium, and nitride, namely, bi-elements of gallium and nitride or tri-elements of indium, gallium nitride, which have a central wavelength of 460 ± 2 nm in this study. Presently, the blue LED is moving forward as the high power with greater luminance option. The blue LED is the shortest wavelength among the visible LEDs and illustrates the most potent energy. On the other hand, the white LED is made of blue LED plus yellow phosphors (Y3Al5O12: Ce) mixed in the optoelectrical package, which is the rich-blue spectrum. This paper presents an automatic optical inspection engaged with a light-emitting diode to detect the thin-film transistor.

Moreover, the spectra of the LEDs of the light sources were measured by a commercial off-the-shelf spectrometer in the spectral range from 380 to 780 nm, manufactured by Steag Eta-Optik GmbH (Borsigstr, Heimberg, Germany).

## 4. Results and Discussion

The experiment captured the image of the electrode pixel on the TFT array by multiple-scan TDI-CCD engaged with light-emitting diode using the white, red, green, and blue light to evaluate the image quality. Furthermore, the optical parameter condition is the same for each light source, such as Equation (2) at *WD* (Working distance), Equation (3) at *DoF* (Depth of field), and *FoV* (Field of view). Furthermore, the binary method of a digital-image implement evaluates the image quality based on the gray level variety.

Among the TFT layers, the third layer’s electrode pixel focuses on manufacturing the source/drain (S/D) metal deposited on the thin film. The voltage of the electrode pixel determines when to switch to the liquid crystal. Thus, the characteristics of the third layer are essential to the manufacturing of the TFT product. However, once any foreign defects adhere to the thin film, the electric signal cannot switch the liquid crystal property. The color rendering on the screen may worsen the efficiency. The chemical vapor deposition (CVD) and plasma-enhanced CVD is a crucial process to the film. Simultaneously, the material with the copper metal surrounded by chromium has a thickness of only a couple of nanometers.

**Figure 3:**
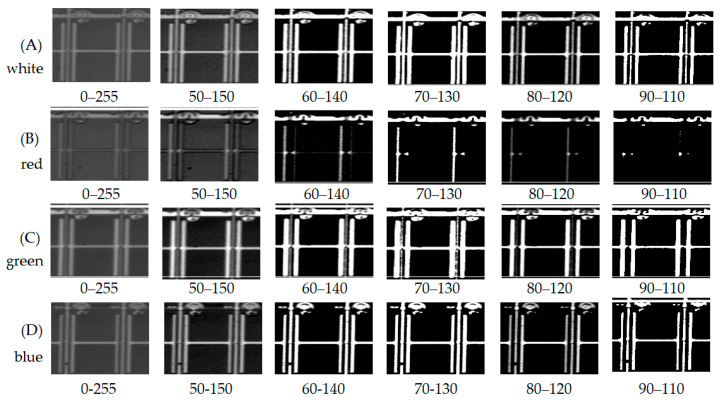
Recognition of the metal electrode on the third layer using white, red, green, and blue LEDs concerning rows (**A**–**D**) at various gray distribution, which is (**A**) white light, (**B**) red light, (**C**) green light, and (**D**) blue light.

The gray distribution illustrates the acquired image by the white, red, green, and blue LEDs in Figure 3. The interval of the gray level introduces six grades, which descends from 0–255 (difference 255), 50–150 (difference 100), 60–140 (difference 80), 70–130 (difference 60), 80–120 (difference 40), and 90–110 (difference 20). The row of Figure 3A’s white light indicates the apparent intensity that the gray value differs from 255, 100, 80, 60, 40, and 20, accordingly. The task arranges the acquired image as Figure 3, a matrix of 4 × 6. Consequently, the matrix of 1 × 6 (90–110) is the raw clearest in A. The row has the smallest gray level and indicates the most contrast between black and white. At the same time, the gray level improves image recognition.

As the white LED light is rich-blue mixed with a yellow phosphor, the spectrum emits the visual wavelength from 380 to 680 nm. Moreover, the wavelength of 380–480 nm distributes in the blue light, i.e., some ultraviolet light in the range.

Figure 3B indicates the most impoverished image, inferior sharp and less contrast through column (B). Figure 3C shows fog image, less intensity, and contrast through column (C). Figure 3D indicates the cleanest image, greater sharpness, and difference through column (D). Compared with the queue (A), (B), (C), and (D), (A) and (D) indicate that both white light and a blue light indicate a precise image. Even so, the blue LED outperformed the other lights.

**Figure 4:**
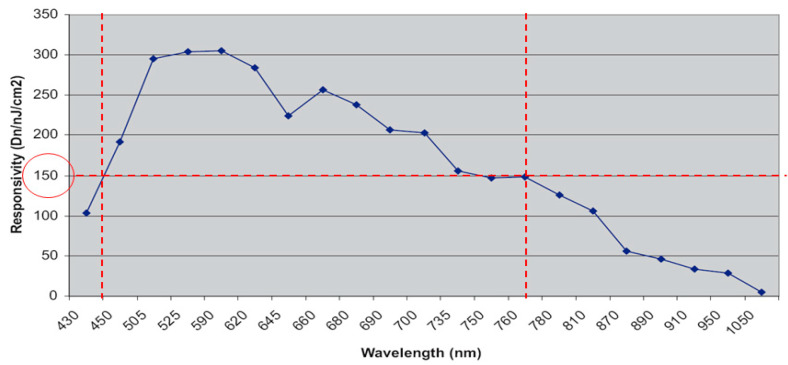
The responsibility of the TDI-CCD (time delay and integration-charge couple device) illustrates spectral distribution.

On the other hand, the photosensor TDI-CCD sensibility adopts an 8-bit data format in which the gray value is between 0 and 255, representing the range of light intensity for each pixel. In contrast, the device’s dynamic range states the responsibility based on the digital number per unit of light intensity, typically expressed in Dn/nJ/cm^2^, where light intensity is described in nanojoules per square centimeter. Figure 4 has a spectral distribution shown as a red mark in the region. Simultaneously, the area is between 150 and 300 Dn/nJ/cm^2,^ and the wavelength is from 450 to 770 nm for the most sensitive spot.

The task takes the binary method of the gray level to analyze the photo contrast. As a result, the image of the 90–110 is the sharpest in both white and blue LED. In contrast, the red LED has the worst intensity because it is far from the short wavelength. The green LED image is not as apparent as the white LED. However, the blue LED indicated better sharpness due to its short wavelength. Also, the chromium enhances the photosensor to image recognition.

Although the spectrum of the red LED and green LED is located in the photosensor’s central sensibility area, the image quality is still too poor to identify the metal electrode even using the binary gray level. The white (rich-blue) and blue LED demonstrate a high spatial resolution. Both the lights can trigger the photosensor efficiently and effectively.

**Figure 5:**
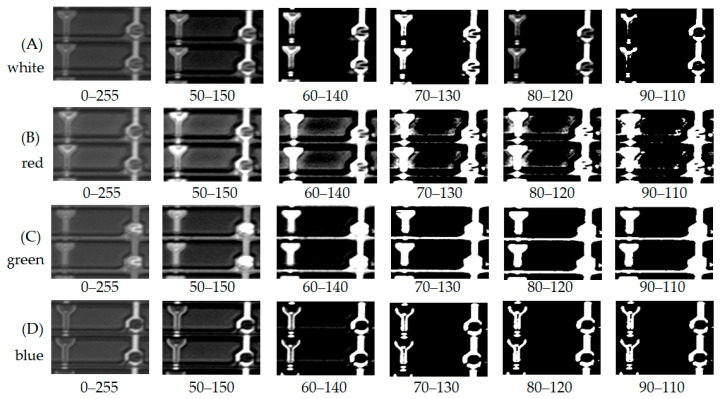
Recognition of the via-hole passivation on the fourth layer using white, red, green, and blue LED detection concerning rows (**A**–**D**) with the various gray distribution concerning (**A**) white light, (**B**) red light, (**C**) green light, and (**D**) blue light.

Figure 5 illustrates the fourth layer on the backchannel passivation that protects the TFT array’s electrical isolation. The backchannel passivation (BP) is a function of the via-hole on the metal layer, a bridge connecting the transistor’s electrical closed-loop. Thus, the manufacturing process is critical for electrical conductibility. The image captured by TDI-CCD concerning the white, red, green, and blue LED is at the same optical condition. The interval of the gray level introduces as previous addresses, which descends from 0–255 (difference 255), 50–150 (difference 100), 60–140 (difference 80), 70–130 (difference 60), 80–120 (difference 40), and 90–110 (difference 20).

In Figure 5A, the white LED indicates that the captured image on the fourth layer is as sharp as possible. However, the red LED on Figure 5B raw has the most inadequate quality of sharpness, indicated by the blurred image and shifts in the pattern. In Figure 5C, green LED illustrates the via-hole with the design as tangled as the image. Among the pictures, Figure 5D shows that the blue LED is a clean image and outperforms other light sources.

Table 1 tabulates the working distance of the optical parameter. The task utilizes the 5.2-μm optical resolution to evaluate this experiment. The magnification of the lens and the functional space is at 80 mm.

Table 2 displays the depth of field while the automatic optical inspection uses the various LED light sources. However, the image is chosen as the optimum height to catch the picture. In the experiment, the optimum acquired image is at 0.67 mm.

The mercury lamp emitted a stable and sustainable spectrum of the line source and always has the optical characteristics to calibrate the spectrometer. It has an electric current to pass the loop on exciting high-pressure mercury vapor to generate a transition between the energy levels. A spectral calibration lamp contains a small amount of mercury to dominate the discharge spectrum. The mercury lamp needs a current of 18 ± 5 mA and has a lifespan of 500 h. This calibrated apparatus adopts the model 6034 pencil lamp, spectral lamp holder, rod mount holder, and power supply, manufactured by Oriel Instrument (Newport, RI, USA).

**Figure 6:**
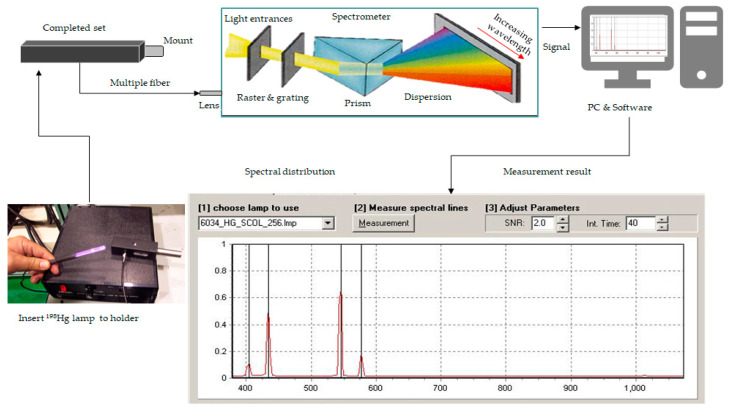
The topology of the completed set by the ^198^Hg pencil lamp to calibrate the spectrometer.

Figure 6 illustrates the calibrated spectrometer’s architecture linking the ^198^Hg discharged lamp, consisting of the ^198^Hg pencil lamp with holder, reflective spectrometer, the PC software, and the spectral measurement result distribution. At first, the ^198^Hg pencil lamp emits the mercury vapor line to the spectrometer. The raster leads the light to enter the prism, making a dispersion distribution by increasing wavelength from blue to red. That wavelength through the electric current excites high-pressure mercury vapor to generate transition. The software identifies the spectrum based on the spectral distribution. The spectrometer detects four distinct sets of emission spectrum lines from the ^198^Hg pencil lamp. Typically, the calibrated criterion defines that a shift of the wavelength is less than 1 pixel for the spectrometer, with a 1-pixel base on the spectrometer capacity. That is, the maximum value subtracts the minimum value, then divides by spectrometer resolution. One pixel is at (780−380)/256 = 1.56 nm. Table 3 tabulates the standard deviation measurement at 0.8 nm for the spectrometer experimentally.

## 5. Conclusions

This paper focuses on various LEDs to detect the electrode pixel on the TFT array using multiple line-scan TDI-CCD and binary digital methods to evaluate the acquired image sharpness and contrast. The gray level distribution is from 0–255, 50–150, 60–140, 70–130, 80–120, and 90–110 to recognize the image sharpness. The wavelength of the white light is from a 380 to 780 nm spectrum, with a rich-blue spectral distribution. The wavelength of the red LED light is at the peak of 660 nm. The green LED wavelength is at the height of 530 nm, and the spectrum of the blue LED is at the peak of 460 nm. The ^198^Hg pencil discharge lamp calibrated the spectrometer is with effective measurement. As a result, the red LED is the most deficient candidate to trigger the photosensor because of its long wavelength. The green LED is not satisfactory as it may be a tangled image that hardly identifies its sharpness in the fourth layer’s via-hole. The white light LED has a rich blue spectrum, which may be a possible candidate in the task. However, the blue LED outperforms the other light sources due to its high spatial resolution and short wavelength.

## Figures and Tables

**Table 1:** The parameter of working distance in the optical evaluation.

Parameter	Value	Unit
Optical resolution	5.2	micrometer (μm)
CCD Pixel Size	5.2	micrometer (μm)
Focused lens	20	millimeter (mm)
Magnification	1	non
Working distance	80	millimeter (mm)

**Table 2:** The parameter of the depth of field (*DoF*) in the optical inspection.

Parameter	Abbreviation	Value	Unit
aperture number	N	2.4	non
the circle of confusion	C	0.012	non
the focal length	f	20	millimeter (mm)
the subject distance	s	80	millimeter (mm)
the depth of field	DoF	0.67	millimeter (mm)

**Table 3:** Tabulations of the calibration of the spectrometer by the ^198^Hg pencil lamp.

Measured (nm)	Lamp (nm)	Difference (nm)
404.08	404.66	−0.58
434.23	435.84	−1.60
545.53	546.07	−0.54
577.31	576.96	0.35
Standard deviation		0.80

## Data Availability

Not Applicable.
